# Hyperprolactinemia in Adults Treated With Anti-psychotic Drugs Attending Tertiary Hospitals in Oman: An Observational Study

**DOI:** 10.7759/cureus.21532

**Published:** 2022-01-23

**Authors:** Mohamed S Al Harthi, Thamra S Al Ghafri, Loai Al Wasify, Salma Al Akhzami, Ahmed AlHarthi, Saud Al Harthi, Nasser Al Sibani

**Affiliations:** 1 College of Medicine, Sultan Qaboos University, Muscat, OMN; 2 Primary Care, Oman Ministry of Health, Muscat, OMN; 3 Laboratory, Al Massara Hospital, Muscat, OMN; 4 Medical Student, Sultan Qaboos University, Muscat, OMN; 5 Medicine, Al Nahdha Hospital, Muscat, OMN; 6 Psychiatry, College of Medicine, Sultan Qaboos University Hospital, Muscat, OMN

**Keywords:** al masarra, oman, sultan qaboos university, antipsychotic, hyperprolactinemia

## Abstract

Introduction: Hyperprolactinemia is a common side effect associated with the use of anti-psychotic medications. This study aimed at exploring the rate of hyperprolactinemia induced by anti-psychotic drugs in adult patients admitted to Sultan Qaboos University Hospital (SQUH) and Al Masarra hospital (AMH). Additionally, factors associated with higher prolactin levels in anti-psychotic patients were explored.

Methods: Bespoke XL sheets on age, gender, region (place of stay), BMI, diagnosis, type of drugs, dose, symptoms, and prolactin levels were recorded from the existing health information system. All adult patients who were on anti-psychotic medication between January 2016 and June 2019 were included. Patients diagnosed with pre-existing endocrine conditions, pregnant females, and those with high prolactin levels at baseline were excluded.

Results: A total of 1103 cases were included in this study of which 34.1% were from the SQUH vs 65.9% from AMH. The mean (SD) age of the study population was 35.6 (12.1), 56.7% were females and 58.7% cases were from Muscat. The common diagnoses were schizophrenia (59.3%) and bipolar affective disorder (14.7%). High prolactin levels existed in 68.3% of the cases from which 59.6% were treated with atypical anti-psychotic drugs. The proportion of cases with high prolactin levels in AMH was significantly different (higher) compared to cases in SQUH (76.9% vs 51.6%, P<0.001).

The most common symptoms were painful breasts (55.2%), galactorrhoea (10.5%), amenorrhea (14.3%) and irregular periods (20.0%). Type of drugs used [haloperidol (typical) vs risperidone (atypical) anti-psychotics (P<0.001)], older vs younger age (P=0.03), and presence vs absence of symptoms (P<0.001) were predictors for the high prolactin levels.

Conclusion: Similar to evidence from the west, results from this study showed a high rate of hyperprolactinemia in adults treated with anti-psychotics. More work is required to standardize anti-psychotic management and monitoring guidelines for psychotic patients across all psychiatric hospitals in Oman.

## Introduction

Hyperprolactinemia is defined as an increase in the level of the hormone prolactin to a level higher than normal in the blood [1]. The elevated level of prolactin has been observed in many neurological, medical, and psychiatric disorders. It is often asymptomatic and might sometimes be associated with a wide variety of side effects. If the prolactin level is high, more tests are required to identify the primary cause. For example, level of the thyroid hormone is often measured to rule out hypothyroidism as a cause of hyperprolactinemia.

Hyperprolactinemia has been reported as a common side effect after use of anti-psychotic medications in patients with psychiatric disease [2,3]. Psychiatric and mental disorders are used interchangeably across the literature. According to a review in 2018, it is estimated that 792 million people in 2017 lived with a mental health disorder. This is slightly more than one in 10 people globally (10.7%) [4]. Psychiatric conditions include schizophrenia, schizoaffective disorder, and bipolar disorder, chronic conditions that require long-term or even lifelong treatment. The first line of treatments of psychiatric conditions are anti-psychotic drugs which are categorized as ‘typical’ or ‘atypical’. The first generation or typical anti-psychotics are the old medications that include chlorpromazine, haloperidol, fluphenazine, and many others. The new second-generation or atypical anti-psychotics include olanzapine, risperidone, quetiapine, and paliperidone [5]. Several side effects have been reported across the literature on both typical and atypical anti-psychotics and many of them have been linked to hyperprolactinemia.

Therefore, determining a baseline level of prolactin before starting an anti-psychotic drug may help clinicians to determine if hyperprolactinemia is actually induced by the medications [6].

The literature suggests that ‘first-generation or typical anti-psychotics have the greatest risk of causing this adverse effect. However, there is also evidence to suggest that ‘second-generation anti-psychotics’ or atypical, particularly risperidone and paliperidone, also increase prolactin secretion [6]. Hyperprolactinemia induced by anti-psychotic drugs has been estimated to occur in up to 70% of patients with schizophrenia [6].

It is postulated that attenuated dopaminergic activities (block D2 receptors) caused by these anti-psychotics are the reason for the development of hyperprolactinemia [7]. Hyperprolactinemia has short- and long-term consequences that can seriously affect the quality of life including, menstrual disturbances, galactorrhoea, sexual dysfunction, gynecomastia, infertility, decreased bone mineral density, and breast cancer. Although many of these are linked to elevated prolactin levels, some, such as breast cancer, require further study. Galactorrhoea can occur in both sexes, but it is more common in women and may occur in up to 57% of those experiencing elevated prolactin levels [2]. Gynecomastia, however, is quite uncommon [8].

Since first-generation anti-psychotics are reasonably less expensive than second-generation anti-psychotics, this may provide clinicians with a reason to consider prescribing first-generation anti-psychotics, increasing the risk of hyperprolactinemia and its consequences. The frequency of anti-psychotic-induced hyperprolactinemia varies, depending on the anti-psychotic agent prescribed [6].

The recommendation to discontinue anti-psychotic therapy in asymptomatic patients with medication-induced hyperprolactinemia is made according to the Endocrine Society Clinical Practice Guideline [9]. Nevertheless, side effects from long-standing hyperprolactinemia, such as osteoporosis and an elevated risk of pituitary adenomas, mandate a re-evaluation of the established anti-psychotic therapy in asymptomatic patients [10]. Several review articles discussed ways to manage hyperprolactinemia induced by anti-psychotics. However, the significance of baseline measurements, follow-up, management plans/guidelines are not consistent across the literature [2].

There is a dearth of studies from Oman on hyperprolactinemia and anti-psychotic medication. The main objective of this study was to describe the rate of hyperprolactinemia induced by anti-psychotic drugs in adult patients with psychiatric conditions admitted to Sultan Qaboos University (SQUH) and Al Masarra Hospitals (AMH). Additionally, the study examined the factors associated with hyperprolactinemia in patients on anti-psychotic medication (typical vs atypical) including gender (male vs female), age (≤35 vs >35), nationality (Omani vs non-Omani), regions of Oman (site of living), diagnosis, comorbidities, and presence of symptoms.

## Materials and methods

This was a retrospective observational study. The study was conducted in two sites, Sultan Qaboos University Hospital (SQUH) and Al Masarra Hospital (AMH), Muscat, Oman. In Oman, the healthcare system is characterized by being free with universal access for Oman citizens and expatriates employed by the Government. AMH is a tertiary care facility with psychiatry services with referrals from and to different regions of the country. SQUH is also a tertiary care teaching hospital located in the capital city, Muscat. It is a general hospital with several specialties, including psychiatry.

Sample size and study population

All eligible patients who were on anti-psychotic medication in the two hospitals between January 2016 and June 2019 were included. After removal of duplicated (n=737) and incomplete records (n=521), 1103 records were included in the assessment.

Inclusion criteria

All patients aged 18 to 60 years who were on an anti-psychotic during the study period with two recorded prolactin levels at least six months prior to the date of data collection. Only patients with a normal prolactin level at baseline were included.

Exclusion criteria

Patients diagnosed with endocrine problems (e.g. pituitary adenoma and hypothalamic disease), pregnant or lactating women at the time of the study, and patients with no baseline prolactin levels and those with insufficient data were excluded from the study. Additionally, all patients on other medications that are known to cause hyperprolactinemia were excluded.

Data collection

Data were extracted from the hospital health information system (Al Shifa 3 plus) and hospital information system (HIS) using a bespoke data collection sheet, which included: number, name and dose of medications, duration of treatment with anti-psychotic medication. The outcome of interest was defined as level of prolactin baseline and last reading recorded at follow up and date of diagnosis. Potential factors associated with hyperprolactinemia included: patient’s age, gender, region, psychiatric diagnosis, comorbidity, and body mass index (BMI). Data were collected by independent researchers who were not aware of study objectives.

Data analysis

Collected data was revised, coded, tabulated and analysed using Statistical Package for Social Sciences (SPSS) version 21 (IBM Corp., Armonk, NY, USA). Mean standard deviation (SD) was calculated for normally distributed data. Frequency and percentage were calculated for non-numerical data.

The dependent variable was levels of prolactin dichotomized as normal or high independently according to the hospitals’ reference ranges [SQUH: reference range = Males: (56-278 Miu/L) and Females: (71-566 Miu/L) vs Al Masarra: reference range = Males: (86-324 uIU/ml) and Females: (102-496 uIU/ml)]. Chi-square test was applied to find factors associated with hyperprolactinemia in patients with anti-psychotic treatment. Studied factors were categorised as: gender (male vs female), age (35 vs >35), nationality (Omani vs non-Omani), regions of Oman (site of living), diagnosis, comorbidities, and presence of symptoms. P-value was used to indicate the level of significance, where p < 0.05 was considered as significant.

## Results

Population characteristics

A total of 1103 cases were included in this study of which 34.1% were from the SQUH vs 65.9% from AMH (Table 1). Mean age for the total population was 35.6 (12.1) [33.6 (11.1) (SQUH) vs 36.6 (12.2) (AMH)]. There were 56.7% females across the two hospitals [65.2 (SQUH) vs 52.3% (AMH)]. A majority, 90.6%, of the cases were Omanis and 58.7% of the cases were from Muscat vs 17.8% from Dakhelya regions.

**Table 1:** Patients’ characteristics in SQUH and AMH hospitals including socio-demographic data, anti-psychotic treatment, prolactin levels, symptoms, and change in prolactin levels *Prolactin levels [Sultan Qaboos University Hospital (SQUH) reference range = Males: (56-278 Miu/L) and Females: (71-566 Miu/L) (Al Masarra Hospital (AMH) reference rage = Males: (86-324 uIU/ml) and Females: (102-496 uIU/ml)].

Parameter	Total population n=1103	SQUH n=376(34.1%)	AL Masarra n= 727(65.9%)
Sociodemographic			
Age mean (SD), IQR	35.6(12.1), (27,34,43)	33.6(11.1), (24 ,32 ,41)	36.6(12.5), (29 ,35 ,44)
Gender			
Male	478(43.3)	131 (34.8)	347 (47.7)
Female	625(56.7)	245 (65.2)	380 (52.3)
Nationality			
Citizen	999(90.6)	320 (85.1)	679 (93.4)
Resident	104(9.4)	56 (14.9)	48 (6.6)
(Governorates) Place of stay			
Muscat	648(58.7)	227 (60.4)	421 (57.9)
Dakhelia	196 (17.8)	60 (16)	136 (18.7)
Dhahira	99 (9.0)	41 (10.9)	58 (8.0)
Batinah	72 (6.5)	22 (5.9)	50 (6.9)
Wusta	28 (2.5)	8 (2.1)	20 (2.8)
Sharqia	24 (2.2)	9 (2.4)	15 (2.1)
Dhofar	16 (1.5)	4 (1.1)	12 (1.7)
Buraimi	20 (1.8)	5 (1.3)	15 (2.1)
Diagnosis			
Schizophrenia	654 (59.3)	207 (55.1)	447 (61.5)
Bipolar affective disorder (BAD)	162 (14.7)	75 (19.9)	87 (12.0)
Obsessive compulsive disorder (OCD)	15 (1.4)	13 (3.5)	2 (0.3)
Attention deficit hyperactivity disorder (ADHD)	9 (0.8)	9 (2.4)	0
Autism spectrum disorder (ASD)	3 (0.3)	3 (0.8)	0
Other	260 (23.6)	69 (18.4)	191 (26.3)
Treatment			
Typical	134 (12.1)	29 (7.7)	105 (14.4)
Atypical	657 (59.6)	288 (76.6)	369 (50.8)
Both	303 (27.5)	50 (13.3)	253 (348)
Other	9 (0.8)	9 (2.4)	0
Prolactin level*			
Value(SD)	1244.1(1358.4)	392.2(765.6)	1533.6(1500.7)
IQR	(319,951,1476)	(28,68,490)	(499,1073,1998)
Minimum value	Minimum 15	Minimum 15	Minimum 85
Maximum value	maximum 9777	maximum 9199	maximum 9777
High	753 (68.3)	194 (51.6)	559 (76.9)
Normal	350 (31.7)	182 (48.4)	168 (23.1)
Hyperprolactinemia symptoms			
Asymptomatic	975 (88.4)	346 (92.0)	629 (86.5)
Symptomatic	128 (11.6)	30 (8.0)	98 (13.5)
Body mass index	28.9(10.4)	29.2(5.0)	28.8(12.3)
Prolactin levels (change)			
Increased	726 (65.8)	178 (47.3)	548 (75.4)
Decreased	247 (22.4)	198 (52.7)	49 (6.7)
No change	130 (11.8)	0	130 (17.9)

Diagnosis and treatment

The common diagnoses identified in the study were schizophrenia (59.3%) followed by bipolar affective disorder (BAD) (14.7%). Most cases, 59.6%, were treated with atypical anti-psychotic drugs in both hospitals. However, results showed that the proportion of cases in AMH who were on typical anti-psychotics (14.4%) was higher than those in SQUH (7.7%) and the difference in this proportion was significant (P<0.001).

Rate of high prolactin levels

**Figure 1:**
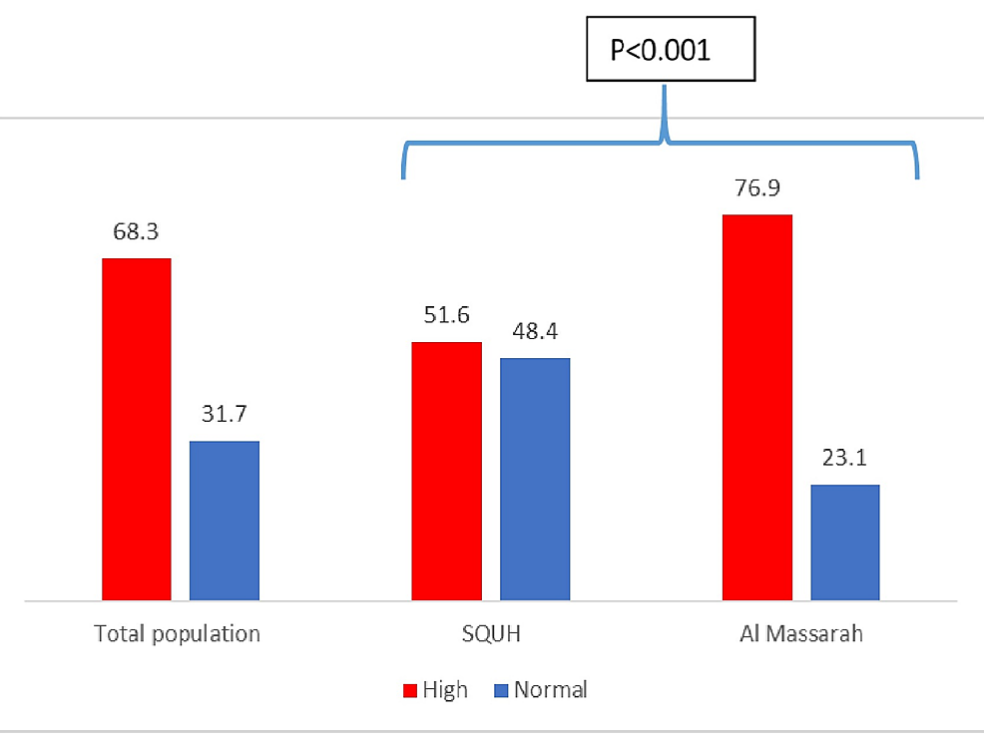
Levels of high vs normal prolactin levels in total population, Sultan Qaboos University Hospital (SQUH) and Al Masarra hospitals

More than half of the cases had high prolactin levels (68.3%) (Figure 1). The proportion of cases with high prolactin levels in AMH was significantly different (higher) compared to the proportion of high prolactin levels in SQUH (P=76.9% vs 51.6%, P<0.001). Mean prolactin level in AMH was 1533.6 (1500.7) vs 392.2 (765.6) in SQUH.

**Figure 2:**
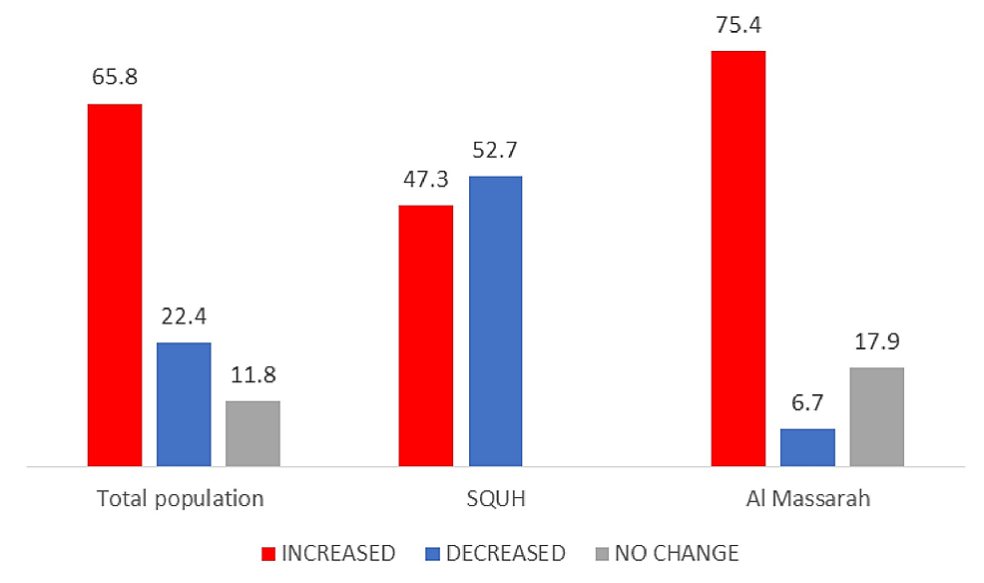
Percentage of prolactin levels that increased, decreased or with no change at last visit from baseline (before starting anti-psychotic drugs) SQUH: Sultan Qaboos University Hospital

Analysis on the change in prolactin levels in the last visit from baseline showed that the proportion of cases who had an increase in their prolactin levels was significantly higher in AMH hospital 75.4% compared to 47.3% in SQUH (P<0.001) (Table 2, Figure 2). 

**Table 2:** Factors associated with high prolactin levels in adults treated with anti-psychotic drugs AMH: Al Massara Hospital; SQUH: Sultan Qaboos University Hospital *Prolactin levels [SQUH reference range = Males: (56-278 Miu/L) and Females: (71-566 Miu/L) (Al Masarra reference rage = Males: (86-324 uIU/ml) and Females: (102-496 uIU/ml)].

Parameters		Prolactin levels*		P-value
		Normal n=350 (%)	High n= 753(%)	
Setting	AMH	168 (23.1)	559 (76.9)	<0.001
	SQUH	182 (48.4)	194 (51.6)	
Gender	Female	202 (32.3)	423 (67.7)	0.3
	Male	148 (31.0)	330 (69.0)	
Age	≤35	208(50.2)	206(49.8)	0.03
	>35	273(39.6)	416(60.4)	
Nationality	Resident	33 (31.7)	71 (68.3)	0.5
	Citizen	317 (32.0)	682 (69.0)	
Typical/Atypical anti-psychotic drugs	Typical	37 (27.6)	97 (72.4)	<0.001
	Atypical	259 (39.4)	398 (60.6)	
	both	49(16.2)	254 (83.8)	
	other	5(55.6)	4 (44.4)	
Regions	Muscat	206 (31.8)	442 (68.2)	0.3
	Dakhelia	56 (28.6)	140 (71.4)	
	Dhahira	40 (40.4)	59 (59.6)	
	Batinah	16 (22.2)	56 (77.8)	
	Wusta	12 (42.9)	16 (57.1)	
	Sharqia	10 (41.7)	14 (58.3)	
	Dhofar	5 (31.3)	11 (68.8)	
	Buraimi	5 (25.0)	15 (75.0)	
Symptoms	Asymptomatic	329 (33.7)	646 (66.3)	<0.001
	Symptomatic	21 (16.4)	107 (83.6)	

**Figure 3:**
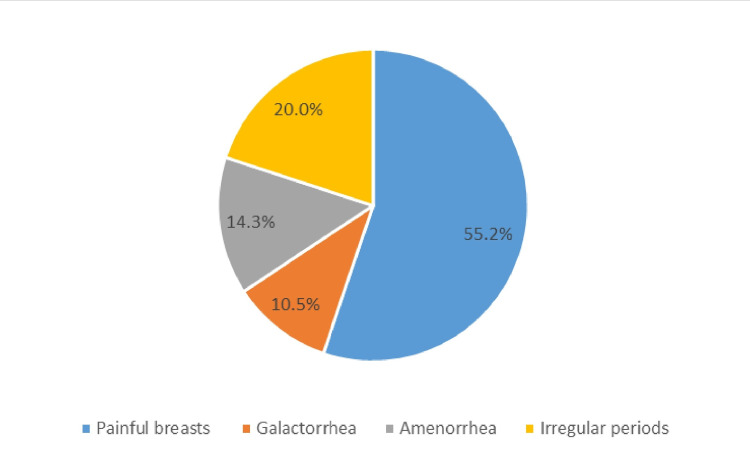
Percentage of the most common presented symptoms in hyperprolactinemia in adults treated with anti-psychotic drugs

Additionally, higher prolactin levels were found in cases that received typical and a combined (typical and atypical) anti-psychotic drug (P<0.001). Most common anti-psychotic drugs were risperidone (42.5%), haloperidol (13.2%), olanzapine (10.2%), fluphenazine (9.9%) and other mixed regimens (24.2%). Significantly, higher prolactin levels were associated with higher doses of haloperidol (typical) (P=0.003) and risperidone (atypical) (P=0.01). Also, the proportion of high prolactin was significantly higher in symptomatic vs asymptomatic cases (P<0.001) (Table 2). The most common symptoms in the general study population were painful breasts (55.2%), galactorrhoea (10.5%), amenorrhea (14.3%), and irregular periods (20.0%) (Figure 3). In females, 62% reported galactorrhoea whereas 59.3% of males reported painful breasts.

The proportion of older cases with high prolactin was significantly higher compared to younger cases (P=0.03). Other studied factors such as gender, nationality, regions, and BMI were not associated with high prolactin levels.

## Discussion

The current study looked at rate of prolactin levels in patients on anti-psychotic drugs in two major psychiatric hospitals in Oman, Muscat (SQUH and AMH). The study included 1103 cases (34.1% from SQUH vs 65.9% from AMH). Hyperprolactinemia was found in 68.3% of the studied cases (51.6% in SQUH vs 76.9% in AMH). Similar to studies in the literature, anti-psychotic-induced hyperprolactinemia has been estimated to occur in up to 70% of patients with schizophrenia [6].

Typical anti-psychotics (chlorpromazine, and haloperidol) commonly induce elevations above the normal range, as do some of the newer anti-psychotics, most notably risperidone and amisulpride. Studies by Wieck and Haddad estimated that 60% of women and 40% of men treated with anti-psychotics develop a prolactin level above the normal range, possibly 10-fold increases from baseline [11,12].

Hyperprolactinemia was significantly higher in cases from AMH compared to SQUH. Reasons for this could be attributed to the high use of typical anti-psychotic drugs in AMH compared to SQUH (7.7% in SQUH vs 14.4% in AMH). Consistently, there was an increase in prolactin levels from baseline readings in more cases from AMH (75.4%) compared to SQUH cases (47.3%). The strongest reported predictors of hyperprolactinemia are the type and higher doses of the anti-psychotic drug prescribed [6]. In the case of types of anti-psychotics, the current study confirms findings reported by Inder and Castle that haloperidol, a typical drug, gave rise to the greatest prolactin level increase. Also, risperidone was among the highest elevators of the atypical anti-psychotics. On the other hand, olanzapine and quetiapine had less effect on elevating prolactin levels [6].

In addition, older age was equally associated with high prolactin levels in the current study. Studies have reported similar findings as complications induced by anti-psychotics increase with age [13,14].

Moreover, the prevalence of hyperprolactinemia in premenopausal women was reported to be higher than in men and higher than in postmenopausal women [13]. Despite slight increase in hyperprolactinemia among females (67.7%) vs males (69.0%), this gender difference was not significant in the current study. However, several studies have suggested that young females were at higher risk for hyperprolactinemia [15-17].

The presence of symptoms was significantly associated with high prolactin levels. This finding could be linked to a longer duration of illness. Duration of illness/psychosis in patients treated with anti-psychotic medication appeared to be associated with prolactin levels [18].

Unfortunately, there was a lack of data in the health information system from both the studied hospitals on the workout/investigations performed to exclude other causes of hyperprolactinemia [19].

Clinical implication of the results from this study

There is currently no consensus on whether screening for hyperprolactinaemia in the management of psychotic patients is useful, whereas it appears that pre-treatment screening may be helpful [20]. In Oman, this practice needs to be endorsed.

The risks and benefits of discontinuing or changing an anti-psychotic regimen versus the possibility of psychiatric illness relapse must be studied and weighed [10]. Side effects (such as galactorrhoea, gynecomastia, oligomenorrhea, amenorrhea, infertility, sexual dysfunction, and osteoporosis) and the level of patient distress must be monitored. However, monitoring of prolactin is “generally not recommended” in clinical guidelines [21]. Thus, an elevated prolactin level will only be detected after inquiring about the patient’s side effects to medication. Thus, it is important to ask about side effects as patients may not declare sensitive or personal information if not asked. Even though no guidelines exist for routine prolactin monitoring, some symptoms, routine monitoring of patients taking anti-psychotic regimens may be implemented [22].

Strengths and limitations of this study

The sample size can be considered as fair and results from this study can be generalized. Information obtained from this study can be utilized to standardize anti-psychotic treatment guidelines at a national level. Additionally, this study provides fundamental information on the capacity building activities required for health care providers on the side effects of the anti-psychotics, namely hyperprolactinemia.

Treatment with anti-psychotic medication can be associated with hyperprolactinemia, which may be asymptomatic or associated with a wide variety of side effects. Before anti-psychotic therapy, recording a baseline prolactin level may help in determining whether a patient’s elevated level is due to medication-induced hyperprolactinemia or other causes. One must conduct a thorough work-up of hyperprolactinemic patients to rule out other causes, and then carefully consider the risks and benefits of maintaining the patient on the therapeutic anti-psychotic regimen, altering the dose, changing the medication, or adding other medications to specifically address adverse effects. It is highly recommended to monitor prolactin levels in patients taking anti-psychotics, despite the lack of consensus on the frequency of monitoring procedures. Finally, it seems that the management of hyperprolactinemia induced by anti-psychotics is maximized when it is personalized and tailored to patients’ needs and health status [23].

There were several limitations in this study. Firstly, the two hospitals used different references for prolactin levels due to differences in calibrations. Secondly, the present study was not equipped to tease out the baseline level of prolactin. However, it is reassuring that the results were similar to the currently published papers in the literature. Thirdly, there were no standardized guidelines on how to manage side effects of the anti-psychotic drugs at the national level. Lastly, other side effects were not recorded and risks for cancer (breast, pituitary) were not recorded.

## Conclusions

This is a study that looked at the rate of hyperprolactinemia in adult patients treated with anti-psychotic drugs in SQUH and AMH hospitals. Results showed high rate of hyperprolactinemia in both hospitals. Type of drugs used (mainly typical anti-psychotics), age (older age), and presence of symptoms were significant predictors for the high prolactin levels. Haloperidol and risperidone were highly associated with elevated levels of prolactin. Most cases were asymptomatic in both hospitals. However, the most common symptoms were painful breasts, galactorrhea, amenorrhea and irregular periods. Management and monitoring guidelines are needed to follow patients with such side effects and evaluate the effectiveness of various interventions to reduce side effects and weigh benefits.
